# Comparison of BARD®LIFESTREAM™ covered balloon-expandable stent versus GORE® VIABAHN™ covered self-expandable stent in treatment of aortoiliac obstructive disease: study protocol for a prospective randomized controlled trial (NEONATAL trial)

**DOI:** 10.1186/s13063-022-06332-7

**Published:** 2022-05-12

**Authors:** Yuhan Qi, Jiarong Wang, Jichun Zhao, Bing Huang, Fei Xiong, Xiaojiong Du, Xiyang Chen, Qiang Guo, Tiehao Wang, Ding Yuan

**Affiliations:** 1 Department of Vascular Surgery, West China Hospital, Sichuan University, 37 Guo Xue Alley, Chengdu, 610041 Sichuan Province China; 2 West China School of Medicine, Sichuan University, Chengdu, China

**Keywords:** Randomized controlled trial, Covered balloon-expandable stent, Covered self-expanding stent, Aortoiliac obstructive disease

## Abstract

**Background:**

Covered stent has become one of the mainstream therapies for aortoiliac obstructive disease (AIOD), with a higher patency rate than bare metal stent. Covered balloon-expandable (CBE) stent can be placed more accurately with higher a radial support force, while covered self-expanding (CSE) stent has greater elasticity and higher trackability. However, there is no level I evidence regarding the comparison safety and efficacy between the CSE stent and CBE stent in AIOD to date. Therefore, this study aims to compare the efficacy and safety of CBE stent (BARD®LIFESTREAM™) and CSE stent (GORE® VIABAHN™) in AIOD.

**Methods:**

This trial is a prospective, single-center, parallel, noninferiority, randomized controlled trial. A total of 106 patients will be enrolled and these patients will be randomized to either the CBE stent group or the CSE stent group. The primary end point of the study is the occurrence of target lesion revascularization (TLR) at 12 months after the intervention.

**Discussion:**

To our knowledge, the ballooN sElf cOver steNt AorToiliAc occuLusive (NEONATAL) trial is the first RCT to compare CBE and CSE stent in AIOD patients. The main aim is to compare the TLR of the target lesion between CBE stent and CSE stent at 12 months post-procedure. The results of clinical trials may contribute to establishing a strategic guideline for choosing the optimal type of covered stent in the treatment of AIOD patients.

**Trial registration:**

Chinese Clinical Trials Registry ChiCTR2100046734. Registered on 27 May 2021

**Supplementary Information:**

The online version contains supplementary material available at 10.1186/s13063-022-06332-7.

## Introduction

Aortoiliac obstructive disease (AIOD) is a common chronic atherosclerotic arterial occlusive disease involving infrarenal aorta or iliac arteries that accounts for approximately one-third of all peripheral artery disease cases and places a high economic burden on treatment [1]. Trans-Atlantic Inter-Society Consensus (TASC) recommends the endovascular approach as the preferred treatment for TASCII A and B aortoiliac lesions instead of open surgery [2]. A related systematic review showed that endovascular treatment was considered valid as open surgical revascularization due to shorter hospitalization and a similar long-term patency rate in TASCII C and D lesions [3, 4]. Furthermore, the recent guideline from the European Society of Vascular Surgery suggested that an endovascular-first strategy may be considered for aorto-iliac occlusive lesions if performed by an experienced team [5]. Regarding device selection in aortoiliac artery interventions, covered stents have a higher class of recommendation in moderate to severe calcified and diffuse lesions than bare metal stents with superior patency rate [6–8].

Covered stents are classified as self-expandable or balloon-expandable, according to the mechanics of stent deployment. Technically, self-expandable stents have greater elasticity, allowing them to maintain their shape after post-dilation and continue to expand as the vessel remodels until they reach the appropriate size. In addition, self-expandable stents are more flexible and trackable; thus, they more easily pass through tortuous vessels. By comparison, balloon-expandable stents have much greater radial support strength, which is especially vital in diffuse, heavily calcified lesions. In addition, balloon-expandable stents can be deployed more precisely with minimal geographic displacement; hence, they are adopted widely in lesions involving aortic bifurcations.

Current evidence has shown that the 1-year restenosis rate and target lesion revascularization rate of self-expandable bare stents are higher than those of balloon-expandable bare stents in AIOD patients [9]. However, the efficacy and safety of covered balloon-expandable (CBE) stents versus covered self-expanding (CSE) stents in the treatment of aortoiliac obstructive disease remain unknown.

Current evidence regarding CBE and CSE stents in AIOD mainly focuses on comparisons with bare metal stents (BMSs). Several studies suggested that patients who received CBE stents had an enduring patency advantage, a lower rate of revascularization, and a similar limb amputation rate compared with those who received BMS [6, 8]. Additionally, another study showed that CBE stents were superior to bare metal balloon-expandable stents for the treatment of common iliac artery (CIA) disease at 3 years regarding primary patency, assisted patency, and secondary patency [10]. However, another study demonstrated that the use of CSE stents has similar early and midterm outcomes compared with BMS in severe iliac lesions, while CSE seemed to have higher midterm patency than BMS only in TASC D subjects [11]. As there is no direct comparison between CSE and CBE stent in AIOD patients, no definitive conclusion can be drawn regarding which type of stent is superior.

The Gore Viabahn endoprosthesis (W. L. Gore and Associates, Flagstaff, Ariz) and LifeStream covered iliac stent (Bard Peripheral Vascular, Inc, Tempe, Ariz) represent one of the most widely used CSE and CBE stents, respectively. This prospective, single-center, parallel, noninferiority, randomized controlled trial aims to compare the safety and efficacy of CSE stents and CBE stents in AIOD patients, with a special focus on midterm and long-term freedom from target lesion revascularization (TLR).

## Methods/design

### Ethics and dissemination

This study was approved by the Ethics Committee on Biomedical Research, West China Hospital of Sichuan University (approval number: 2021-212) on 11 March 2021. The trial was registered in the Chinese Clinical Trial Registry (registration number: ChiCTR2100046734) on 27 May 2021.

### Protocol amendments

In our trial, simple size may be adjusted based on TLR at 12 months post-procedures, which will be notified to the Ethics Committee on Biomedical Research, monitoring committee, and the upstream authorities. Other non-substantial amendments will not be notified to the monitoring committee and the upstream authorities, but will be recorded by researcher. Update informed consent forms will be sent to new patients after amendments, and all researches will be informed correspondently.

### Data access

Project principal investigators, ethics committee, and monitoring committee will have access to the final trial dataset and all data information will be de-identified. Furthermore, independent institution will be contacted for monitoring and quality assurance during the conduct of the study.

### Post-trial care

There is no underlying harm for trial participant when LIFESTREAM or VIABAHN stent placed. After the procedure, patients will be provided with guidance on specialized treatment protocols and dressing changes.

### Study setting

**Fig. 1:**
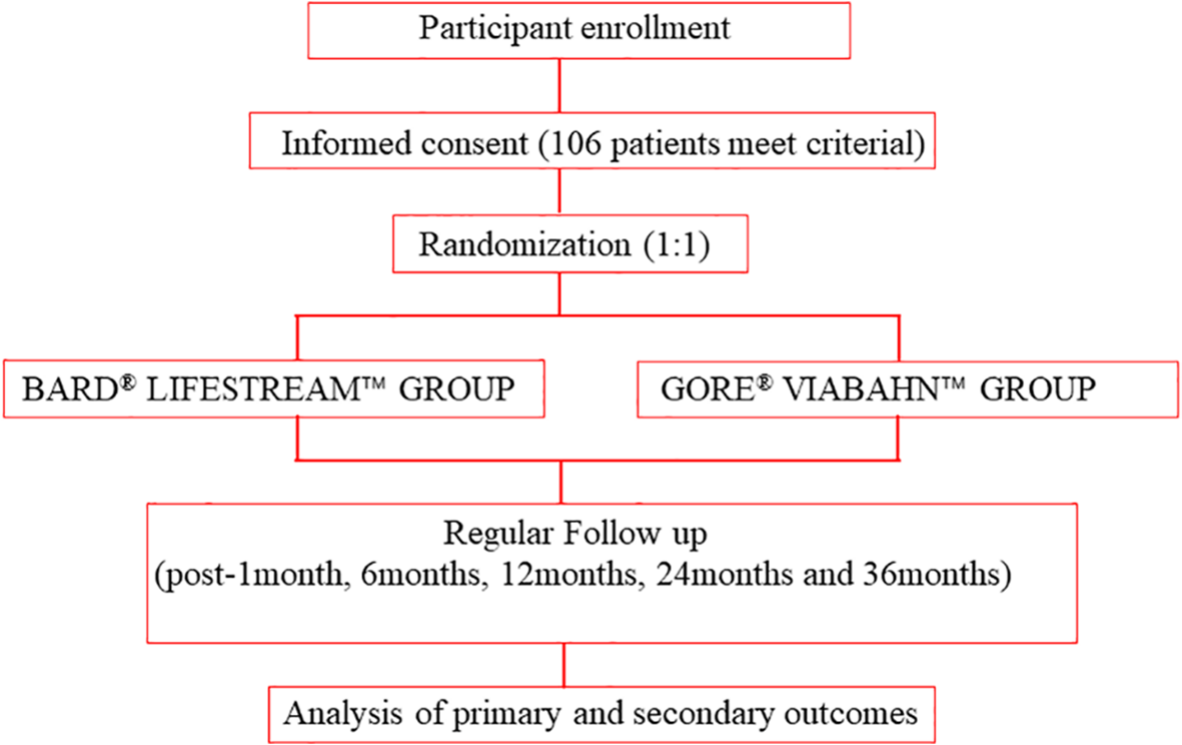
The procedure of screening, randomization, and follow-up of patients in the trial

The NEONATAL trial is a prospective, single-center, parallel, noninferiority, single-blind, randomized controlled trial (allocation ratio 1:1), planned to be conducted in West China Hospital, Chengdu, China. The present protocol was written according to the Standard Protocol Items: Recommendations for Interventional Trials (SPIRIT) 2013 Statement for study protocols of clinical trials [12]. Patient-centered care was involved in the design of our protocol. We conducted a preliminary survey in patients to investigate the outcomes they care most and the follow-up methods that they prefer. The SPIRIT flow diagram is shown in Fig. 1, and the SPIRIT Checklist is added in Additional file 1. Table 1 shows the schedule of enrollment, interventions, and assessments.

**Table 1:**
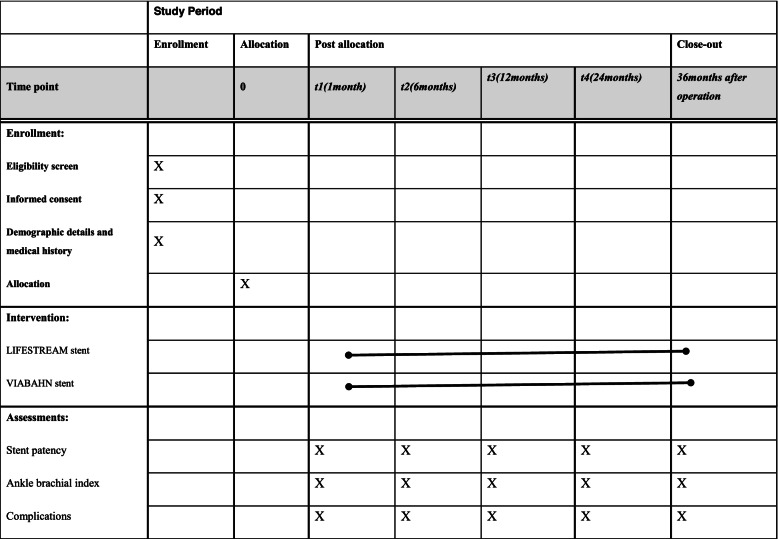
The schedule of enrollment, interventions, and assessments

### Eligibility criteria

Patients at least 18 years old who are diagnosed with AIOD (Rutherford classification from 2 to 6) will be screened for study recruitment. The inclusion and exclusion criteria are shown in Table 2.

**Table 2:** Inclusion criteria and exclusion criteria

Inclusion criteria	Exclusion criteria
The participants meeting the following criteria will be included: (1) The subject is a male or non-pregnant (or no plan to become pregnant during study) female ≥ 18 years old with an expected lifespan sufficient to allow for completion of all study procedures. (2) Symptomatic peripheral artery disease with moderate to severe claudication (Rutherford score of 2 to 3); chronic CLI with resting ischemic pain (Rutherford score of 4); chronic CLI with ischemic ulcers (Rutherford score of 5 to 6). (3) Subjects diagnosed with aortoiliac obstructive disease (Trans-Atlantic Inter-Society Consensus, TASC II, TASC II A-D). (4) The target lesion(s) can be successfully crossed with a guide wire and pre-dilated with an appropriately sized percutaneous endovascular angioplasty (PTA) balloon. (5) The subject provides informed consent using an informed consent form (ICF) that is reviewed and approved by the Ethics Committee (EC).	The participants meeting the following criteria will be excluded: (1) The subject is asymptomatic, has mild claudication described as Rutherford Category 0 (asymptomatic) or 1 (mild claudication). (2) The subject has a vascular graft previously implanted in the native iliac vessel. (3) The subject has an abdominal aortic aneurysm (AAA) contiguous to the iliac artery target lesion(s) or subject has a pre-existing target iliac artery aneurysm or perforation or dissection of the target iliac artery. (4) The subject has a serum creatinine ≥ 2.5 mg/dl or is currently on dialysis. (5) The subject has a known uncorrectable bleeding diathesis or active coagulopathy (platelet count< 80,000/μL). (6) The subject has intolerance to the antiplatelet, anticoagulant, or thrombolytic medications. (7) The subject has a known allergy or sensitivity to stainless steel (i.e., Nickel), ePTFE or contrast media. (8) The subject suffered a stroke, or transient ischemic attack (TIA) within 3 months prior to the procedure.

### Allocation and implementation of interventions

Prior to the endovascular intervention, patients will be enrolled, and informed consent will be obtained from all participants by surgeons. The included patients were randomly allocated at a 1:1 ratio to the CBE stenting group or the CSE stenting group using Statistical Analysis System (SAS, version 9.4) by a biostatistician within sex and 5-year age groups in blocks of ten, and stratification was performed by numbers of treatable legs. For patients who required intervention in bilateral iliac arteries, randomization was performed according to the treated arteries. The sealed, opaque, consecutively numbered envelopes were used to keep the allocation code for each participant, which will be kept unopened by an independent research assistant. The nurse research coordinator in the operating room will prepare the envelopes and inform the surgeon of the allocation results before intervention. Both nurse research coordinators and biostatisticians were not involved in the perioperative management of patients. The outcome assessors and data analysts will be blinded to the interventions.

### Details of intervention

The included patients will receive the same perioperative management, including antiplatelet drugs, statin usage, and blood controls. Both common femoral arteries and sometimes brachial artery will be used for access. Whether to adopt percutaneous or cutdown access route will be determined by the surgeons. After obtaining arterial access, 0.5 mg/kg of unfractionated heparin will be administered. A fluor opaque ruler will be used to record the location and extent of the lesion. Both intraluminal and subintimal recanalization of iliac lesions will be allowed. After the successful passage of the guidewire, the target lesion will be predilated with downsized balloon in CBE group, and with equal-sized balloon in CSE group. During actual intervention, the allocated choice of covered stents may be modified according to intraoperative findings, for instance, the length of lesions did not match the stents. Multiple stents are required when needed and multiple stent edges should overlap by at least 10 mm. Furthermore, additional bare metal stents can be added in both groups, according the discretion of surgeons. Post-dilation balloon will be performed within the stented segment, with less than 10% oversizing. Final angiography will be performed after the intervention in both groups, with the same angles and magnifications as the baseline angiography.

Postoperative management include a dual antiplatelet regimen for 3 months, involving 100 mg of aspirin once a day plus 75 mg of clopidogrel once a day. Clopidogrel is not required if a participant is taking warfarin; other direct thrombin inhibitors, factor Xa inhibitors, or low molecular weight heparin will be used according to the physician’s recommendation. Anticoagulant therapy will be discontinued when other surgical procedures are needed, and then, the participant will resume anticoagulant therapy as soon as possible after the surgery. Drug side effects will be checked and monitored at outpatient visit or telephone follow-up. According to the comprehensive surgeon’s recommendation, drug therapy will be discontinued. Clinical follow-up will be planned at 1, 6, 12, 24, and 36 months to evaluate clinical outcomes.

### CBE and CSE stent

The CBE stent used in our trial is BARD® LIFESTREAM™ Covered Stent, a permanent stent used to maintain patency of the common or external iliac artery. The stent is a balloon-expandable stent with expanded polytetrafluoroethylene (ePTFE) encapsulated between two layers (inner and outer). The implant is pre-mounted on an over-the-wire balloon catheter that acts as the delivery system. The configurations of BARD® LIFESTREAM™ covered stent offered 16, 26, 37, 38, and 58 mm in length and 5 to 12 mm in diameter. The stents can be overlapped if the target lesion exceeds the longest available length of stent.

GORE® VIABAHN™ self-expandable covered stent is the only CSE stent adopted in our trial; it consists of two main components: an intravascular covered stent and a catheter-based delivery system. The stent consists of a self-expanding nickel-titanium alloy stent combined with an ePTFE intracavity coating, which is offered in lengths of 25, 50, and 100 mm and in diameters of 5 to 11 and 12 mm in the configurations.

### Follow-up and outcomes of interest

Primary outcome and secondary outcomes are set based on patient-centered outcomes. All included patients will be followed up in outpatient clinics using questionnaires at 1 month, 6 months, 12 months, 24 months, and 36 months after the initial intervention. Patients who are unable to return will be followed up on telephone. Duplex ultrasound will be tested routinely at 1, 6, 12, 24, and 36 months post-index procedure; CTA will be routinely tested at 12 and 36 months post-index procedure; and if duplex US suggests restenosis at the target lesion, a further CTA examination will be performed to assess the stenosis. If the restenosis is more than 50% and the patient’s symptoms are worse (e.g., shorter claudication distance or exacerbation of pain), reintervention will be carried out. Patients who are unable to return to clinic will be suggested to undergo computed tomography angiography (CTA) or duplex ultrasonography in local hospital. Images and medical records will be sent to investigators online. Trained outcomes assessor will collect the outcomes of interest, blinded to the intervention groups.

The timepoint for the primary outcome of this study is 12 months. Patients are defined as dropout if they meet the following criteria: (1) lost to follow-up for less than 12 months post-intervention: a subject is considered loss to follow-up if cannot be contacted via fifth attempts by telephone and WeChat message. (2) Withdrawal consent to participation of the study: all participants have the right to withdraw from the study at any time for any reason.

#### Primary outcome

Target lesion revascularization (TLR) of the target lesion(s) assessed at 12 months after the intervention (TLR is defined as the first revascularization procedure (e.g., percutaneous transluminal angioplasty (PTA), atherectomy, and catheter-directed thrombolysis)) when the primary patency is lost.

#### Secondary outcomes

The following secondary endpoints of this study are collected at 1, 6, 12, 24, and 36 months post-index procedure:

(1) Stent and/or surgery related mortality; (2) all-cause mortality; (3) amputation-free survival; (4) acute limb ischemia rate; (5) major adverse cardiovascular and cerebrovascular events (MACCEs); (6) target vessel revascularization (TVR) (TVR is defined as the first revascularization procedure (e.g. PTA, stenting, surgical bypass, etc.) in the target vessel(s)); (7) primary patency (the loss of primary patency is defined as target lesion(s) is determined to be > 50% stenosis or TLR/TLR needed in follow up); (8) primary assisted patency (the loss of primary assisted patency is defined as target lesion(s) is determined to be > 50% stenosis after TLR); (9) secondary patency (the loss of secondary patency is defined as target lesion(s) is determined to be > 50% stenosis after endovascular procedure or surgical bypass TVR); and (10) stent-related complication (e.g., stent kinking or fracture).

### Data collection and management

Two investigators will use a pre-identified and unified case report form (CRF) to record the data of included patients independently. Both received training for data collection. Demographics, comorbidities, and prescriptions of cardiovascular drugs were recorded from patient chart. Imaging anatomic parameters were measured from picture archiving and communication system (PACS). Laboratory results were collected from laboratory information system (LIS).

As for data management, two investigators retrieved data from CRF and recorded them in two identical electronic forms, and cross check will be performed to ascertain the accuracy of data. We also set range checks for data values. Importantly, the investigators will be urged to follow up on patients by telephone contacts. Data will be collected in as much detail as possible. Collected data lists will be de-identified and stored in monitoring committee and will be made available to the third-party regulatory body upon request.

### Sample size calculation

We calculated the sample size for this study based on the results of previous studies [4, 13]. As is reported previously in AIOD patients, the 9-month primary patency of CBE stent (BARD®LIFESTREAM™ Stent) was 89.1%, while the 9-month primary patency of the CSE stent (GORE® VIABAHN™ stent) was estimated to be 82.2% based on VIASTAR Trial [14, 15]. In the present study, we hypothesized that the primary patency of CBE stent is not inferior to that of CSE stent; the predetermined noninferiority margin on risk difference scale (δ) is set as 10% between treatment groups. The type I error rate is 0.05, the power of the test is set as 80%, and the randomization ratio is set as 1:1. After accounting for a 15% dropout rate. It was calculated that 53 lesions per group are needed for a total of 106 lesions.

In order to achieve adequate participant enrollment, we will recruit patients by putting up posters or advertising on the Internet, furthermore, we actively recruit patients from outpatient department and emergency department.

### Statistical analysis

R studio Version 1.2.1335 (http://www.R-project.org) and Empower (www.empowerstats.com, X&Y solutions, Inc., Boston, MA) were utilized for statistical analysis. Categorical data are expressed as numbers and rates, while continuous data are expressed as means ± standard deviation (SD) if they are normally distributed, or median (interquartile range [IQR]) vice versa. Student’s *t* test or Mann-Whitney *U* test is used for univariate analysis of continuous data, and the *χ*^2^ test or Fisher’s exact test is used for categorical data. For primary analyses, multivariate logistic regression is adopted to calculate adjusted odds ratios (ORs) and 95% confidence interval (95% CI) for short-term outcomes. Cox proportional hazard regression analysis is used to generate adjusted hazard ratio (HR) and corresponding 95% CI for long-term outcomes. The primary and secondary end points will be analyzed in the modified intention-to-treat analysis, which includes all subjects undergo randomization and subsequent interventions. Sensitivity analyses of primary and secondary endpoints are performed in the per-protocol population, based on the treatment actually received. The noninferiority margin was set at 10 percentage points for the absolute difference between groups. If the standard of noninferiority was reached, the primary and secondary outcomes were subsequently tested for superiority (*P*<0.05). Noninferiority will be considered proven if conclusions drawn from the intention-to-treat and per protocol analyses are consistent.

Further pre-specified subgroup analyses will be carried out in the following subgroups: TASC II C-D lesions versus A-B lesions, occlusion versus stenosis. Adjustment analyses for primary and secondary endpoints were performed based on the Hochberg method [16]. Multiple imputation was used to handle missing data.

### Monitoring

The monitoring committee consists of biostatisticians, surgeons, investigators, and nurses who are not involved in the conduct of this study or have financial and professional interests. Annual monitoring will be conducted by the monitoring committee. The monitoring committee will provide feedback to investigators and vascular surgeons as soon as there are any problems. Ethics committee and monitoring committee will have access to check the collected data and question the rationality of the study protocol before, during, and after the trial. Classical interim analyses will be performed by an independent statistician who will be a person other than the regular study statistician. The trial will be terminated when one treatment group is significantly superior or inferior to the other treatment group. The final decisions regarding study modifications or stop rest with both ethics committee and monitoring committee. All serious adverse events (SAEs) must be reported as soon as possible. The SAE form contains the following information: identification of the subject, attending surgeon, and description of the SAE (event, beginning and duration, severity, outcome, treatment, or interventions taken). To maintain the quality and safety of trial, unblinding occurs only in exceptional circumstances when knowledge of the actual treatment is essential for further management of the subject.

### Auditing

The researcher and trained auditors meet monthly to ensure that study is being conducted according to protocol. Annual auditing reports will be presented in annual monitoring by auditors.

## Discussion

The past three decades have witnessed open surgery shift to endovascular strategies as the preferred treatment for mild-to-moderate-to-severe AIOD. Endovascular therapy is increasingly preferred as the first-line treatment in most AIOD patients. Different stents have different designs (covered stents, bare metal stents, self-expanding stents and balloon-expandable stents) but which type is the best for AIOD patients has yet to be fully elucidated [17].

Previous studies have shown that covered stents can create a smooth hemodynamically favorable lumen and resist hyperplastic ingrowth through BMS interstices; in addition, covered stents can also protect against iliac artery rupture post-dilation [8, 10, 18]. Self-expandable stents have high elasticity and small radial force, which helped to prevent circumferential stress and subsequently enhanced neointimal proliferation. This may be the reason why self-expandable bare metal stents led to a decreased incidence of restenosis at 12 months compared with treatment with balloon-expandable bare metal stents [9]. However, the best choice between CBE stent and CSE stent is left to the interventionalist’s preference and lack of RCT data now, leading to there is no consensus or relevant guidelines on which stents should be used in AIOD.

The Viabahn stent and LifeStream stent represent one of the most widely used CSE and CBE stents, respectively. A previous study showed that Viabahn covered stent can be considered an acceptable patency with low complications during the long-term period in low-risk young AIOD patients [4], especially for complex aortoiliac occlusion lesions [19]. Another study indicated that the LifeStream stent provided satisfactory 9-month clinical outcomes, including a low rate of TLR for the treatment of stenotic and occlusive lesions of the iliac arteries [15]. However, there is no long-term period patency or TLR of the LifeStream stent. Furthermore, there is no cohort or RCT study to compare the efficacy or effect between Viabahn stent and LifeStream stent. The main aim of our present trial is to compare TLR of the target lesion of LifeStream stent with Viabahn at 12 months’ follow-up.

In conclusion, this trial was designed to compare the efficacy and safety of a CBE stent (BARD®LIFESTREAM™) versus a CSE stent (GORE® VIABAHN™) in AIOD patients, hoping to provide level 1 evidence for stent selection in aortoiliac occlusive lesions.

### Trial status

Ethics approval has been granted before submission. The trial is currently in the recruitment phase. Study enrollment began on 1 June 2020. It is estimated that recruitment will be completed by 1 June 2022, with a study completion date of 1 June 2024. Any protocol amendments will be updated in the Chinese Clinical Trials Registry.

## Supplementary Information

**Additional file 1.** Standard Protocol Items: Recommendations for Interventional Trials (SPIRIT) 2013 Checklist: recommended items to address in a clinical trial protocol

**Additional file 2.**
