# Supplementary material for: Comparison of BARD®LIFESTREAM™ covered balloon-expandable stent versus GORE® VIABAHN™ covered self-expandable stent in treatment of aortoiliac obstructive disease: study protocol for a prospective randomized controlled trial (NEONATAL trial)
Source: Trials. 2022 May 12;23:392. doi: 10.1186/s13063-022-06332-7 (PMC9097112; doi:10.1186/s13063-022-06332-7)
Supplement: Supplementary file 2 — Additional file 2. [file 13063_2022_6332_MOESM2_ESM.docx]

Ethics Committee on Biomedical Research, West China Hospital of Sichuan University

Approval of Biomedical Ethics Review Committee of West China Hospital, Sichuan University 2021-212

| Department: Vascular Surgery | | | Name and title of project leader: Professor Ding Yuan | | |
| --- | --- | --- | --- | --- | --- |
| Project Name | Comparison of BARD^®^LIFESTREAM™ covered balloon-expandable stent versus GORE^®^ VIABAHN™ covered self-expandable stent in treatment of aortoiliac obstructive disease: study protocol for a prospective randomized controlled trial | | | | |
| protocol | Version: 1.0 | | | Version Date: 2021-01-29 | |
| Informed Consent Form | | Version: 3.0 | | | Version Date: 2021-03-11 |
| Review Comments:  1. The qualifications of the investigators meet the ethical requirements.  2. The study protocol and informed consent form basically meet the ethical requirements.  Review results: ■ approved □ approved after revision □ revised and re-examined □ not approved □ suspended or terminated the study  Frequency of continuous review: □ 3 months □ 6 months ■ 1year □ Not applicable/NA  Please follow the relevant laws, rules, and regulations in China (Measures for Ethical Review of Biomedical Research Involving Human Subjects, etc.) as well as the WMA Declaration of Helsinki and the CIOMS International Ethical Guidelines for Human Biomedical Research, and conduct clinical trials (research) in accordance with the protocols and informed consent forms approved by the Ethics Review Committee to protect the health and rights of the subjects.  Please strictly enforce the Regulations of the People's Republic of China on Human Genetic Resources Management (State Decree No. 717). The collection, conservation, international cooperation, and export of materials involving human genetic resources of the hospital must apply for administrative permission from the Ministry of Science and Technology of the People's Republic of China. The Department of Clinical Research Management is the human genetic resources management department of the hospital, and the consultation number is 85422851.  In the event of a change of principal investigator during the course of the trial (study), the applicant is requested to submit an amendment review application for any changes to the clinical study protocol, informed consent form, etc.  In the event of a serious adverse event, applicants are requested to submit a serious adverse event report in a timely manner; a detailed follow-up report of the serious adverse event should be submitted as soon as possible after the emergency report.  Please submit annual and periodic follow-up review reports; when any situation arises that may significantly affect the conduct of the trial (study) or increase the risk to the subjects, the | | | | | |
| applicant is requested to submit a written report to the Ethics Review Committee in a timely manner.  If a trial (study) includes subjects who do not meet the inclusion criteria or the exclusion criteria, if a trial (study) is discontinued without withdrawing the subject from the trial (study), if the wrong treatment or dose is given, if a combination of drugs prohibited by the protocol is given, etc., or if there is a breach of ethical principles and norms that may adversely affect the rights/health of the subject or the scientific validity of the study, the sponsor / reviewer / investigator is requested to submit a report of the breach of protocol.  Applicants suspending or early termination of clinical trials (studies), please submit the suspension/termination of trials (studies) report in a timely manner. Complete the clinical trial (study), please submit the closing report for the applicant.  Without ethical review and approval, clinical research cannot be conducted.  This approval is valid for one year, the implementation of the overdue, it is self-revoked.  According to the requirements of the International Committee of Medical Journal Editors (ICMJE), all clinical studies in human subjects and using specimens taken from human subjects should be registered. Investigators who have received ethical approval are requested to register at the China Clinical Research Registry prior to the start of clinical research.  (<http://www.chictr.org.cn>)  Date: 2021-03-23 | | | | | |
